# Morphological changes in *Proteus mirabilis* O18 biofilm under the influence of a urease inhibitor and a homoserine lactone derivative

**DOI:** 10.1007/s00203-014-0952-8

**Published:** 2014-01-31

**Authors:** Grzegorz Czerwonka, Michał Arabski, Sławomir Wąsik, Agnieszka Jabłońska-Wawrzycka, Patrycja Rogala, Wiesław Kaca

**Affiliations:** 1 Department of Microbiology, Jan Kochanowski University in Kielce, Świętokrzyska 15, 25-406 Kielce, Poland; 2 Institute of Physics, Jan Kochanowski University in Kielce, Świętokrzyska 15, 25-406 Kielce, Poland; 3 Institute of Chemistry, Jan Kochanowski University in Kielce, Świętokrzyska 15, 25-406 Kielce, Poland

**Keywords:** *Proteus mirabilis* O18, Biofilm, Urease inhibitor, Interferometry, FT-IR, BHL

## Abstract

*Proteus mirabilis* is a pathogenic gram-negative bacterium that frequently causes kidney infections, typically established by ascending colonization of the urinary tract. The present study is focused on ureolytic activity and urease inhibition in biofilms generated by *P. mirabilis* O18 cells. Confocal microscopy revealed morphological alterations in biofilms treated with urea and a urease inhibitor (acetohydroxamic acid, AHA), as some swarmer cells were found to protrude from the biofilm. The presence of a quorum-sensing molecule (*N*-butanoyl homoserine lactone, BHL) increased biofilm thickness and its ureolytic activity. Laser interferometric determination of diffusion showed that urea easily diffuses through *P. mirabilis* biofilm, while AHA is blocked. This may suggest that the use of urease inhibitors in CAUTIs may by less effective than in other urease-associated infections. Spectroscopic studies revealed differences between biofilm and planktonic cells indicating that polysaccharides and nucleic acids are involved in extracellular matrix and biofilm formation.

## Introduction

Urinary tract infections (UTIs) are commonly caused by *Escherichia coli* and *Proteus mirabilis* strains. Almost 90 % of UTIs are ascending, with bacteria gaining access to the urinary tract via the urethra, first infecting the bladder and then the upper part of the urinary tract (Hryniewicz et al. 2001), leading to serious medical problems. Biofilm formation, swarming motility, and ureolytic activity are virulence factors characteristic of *P. mirabilis* strains (Stankowska et al. 2012). The composition of *Proteus* sp. exopolysaccharide matrix has not been fully determined yet (Rahman et al. 1999). Biofilms are a serious medical problem during catheter-associated urinary tract infections (CAUTIs) due to the blockage of catheters. The majority of patients with recurrent *P. mirabilis* catheter encrustation (62 %) develop bladder stones later on (Jacobsen and Shirtliff 2011). Antibiotic treatment of CAUTIs is accompanied by the use of acetohydroxamic acid (AHA), a urease inhibitor (Morris and Stickler 1998). Being a urea analog, AHA is administered in order to prevent the formation of renal struvite stones by inhibition of the urease activity of *P. mirabilis* strains (Star et al. 1993). In our previous studies, we focused on the process of *P. mirabilis* O18 biofilm formation in the presence of a series of six derivatives of homoserine lactones (AHLs). We studied mixed *P. mirabilis* O18 and *E. coli* biofilms (Stankowska et al. 2012), and it was shown that only one out of six AHLs, that is, *N*-butanoyl homoserine lactone (BHL), influences biofilms formed by *P. mirabilis* O18 strains.

In this study, we examined *P. mirabilis* O18 biofilm formation in the presence of urea, a urease inhibitor (AHA), and BHL. The developing biofilms were assessed by various microscopic and laser interferometric methods.

## Materials and methods

### Bacterial strains and cultivation

The native *P. mirabilis* O18 laboratory strain PrK 34/57 was obtained from the Czech National Collection of Type Cultures. The strain was transformed by plasmid pDsRed2 (Amp^R^) (Stankowska et al. 2012), strain was also tetracycline resistant (tet^R^). The *P. mirabilis* O18 strain was cultivated at 37 °C for 72–96 h without shaking in LB broth (pH 7.0) supplemented with ampicillin or in liquid Christensen medium (pH 6.8) without a phenol red indicator, supplemented with tetracycline (10 μg/mL) to avoid contamination during long-time cultivation. Ureolytic assays were performed on Christensen medium (Stankowska et al. 2008). For biofilm formation process, bacterial strains were inoculated into liquid medium without shaking (37 °C) to obtain logarithmic phase of growth (from 7 to 13 h, depending on the medium used). Culture in logarithmic growth phase was transferred to biofilm formation vessel and cultivated for 72–96 h without shaking.

### Biofilm studies

*Proteus mirabilis* O18 biofilms were grown in 24-well plates on glass coverslips. Strains were grown in LB broth or Christensen medium (*P. mirabilis* culture supplemented with 100 μg/mL of ampicillin) at 37 °C for 96 h without shaking. Culture media for some experiments were also supplemented with acetohydroxamic acid (AHA, Sigma) at a concentration of 200 μg/mL. The coverslips were washed three times with a sterile 10 mM HEPES buffer and stained (live/dead) with BacLight (according to the protocol recommended by the manufacturer, Invitrogen) for 15 min in the dark. Stained coverslips were placed upside down on slides, sealed with nail varnish, and wiped carefully with a cotton swab with ethanol. For live/dead staining, *P. mirabilis* O18 pDsRed2 strain was cultivated on ampicillin-free medium, which resulted in lack of RFP signal. Representative images were then photographed with a confocal microscope (Leica, Heidelberg). Measurements of biofilm biomass were performed by washing with sterile saline in triplicate, staining with crystal violet (0.4 %) for 15 min, and washing again in saline. The washed wells were filled with 95 % ethanol for 15 min, and absorbance was measured at *λ* = 595 nm (Koerdt et al. 2010). Absorbance measurements were performed in six-well sterile plates (Grainer) or 96-well plates (Nunclon) with an Infinite 200 PRO spectrophotometer (Tecan).

### Effects of BHL on biofilm urease activity

Previous studies (Stankowska et al. 2012) show that the optimal concentration of *N*-butanoyl-dl-homoserine lactone (BHL, Fluka) is 10 nM and such a concentration was used in the presented study. Biofilm was formed in six-well plates (Grainer) at 37 °C for 24 h. Planktonic cells were washed away with sterile saline, wells with biofilm were filled with 2 % urea with phenol red, and pH was adjusted to 6.8. Measurement of absorbance at *λ* = 560 nm was performed at 1 min time intervals using an Infinite 200 PRO Reader (Tecan).

### Urease inhibitor (AHA) effects on *Proteus mirabilis* O18 biofilm formation and swarming behavior

The influence of AHA on the *P. mirabilis* O18 strain was tested in 96-well plates (Nunclon, flat bottom). Cells were cultivated for 8 h in Christensen liquid medium, and then transferred to a microtiter plate with an increasing concentration of AHA. After 24 h of incubation, absorbance for planktonic cells was measured at *λ* = 550 nm. Planktonic cells were washed with sterile saline, and the adsorbed biofilm was stained with 0.4 % crystal violet (CV) for 15 min. After that, excess CV was discarded, the wells were washed three times with saline, and 95 % ethanol was added. After 15 min of incubation at room temperature, absorbance was measured at *λ* = 595 nm. Diffusion of AHA through *P. mirabilis* O18 biofilm was measured in an interferometer system.

Swarming motility of *P. mirabilis* was performed on Petri dish with LB agar (1 %, w/v) supplemented with AHA in concentrations: 0, 100, 200, 500, and 1000 μg/mL). Overnight inoculum was diluted 1:100 in fresh LB broth, and 100 μL was added on the centre of Petri dish. Plates were incubated for 24 h in 37 °C.

### Laser interferometry

Biofilm was formed on nucleopore membranes (polymeric nuclear track membranes) with a pore diameter of 0.9 μm. Membranes were purchased from the Joint Institute for Nuclear Research in Dubna, Russia. In preliminary experiments, it was found out that nucleopore membranes do not constitute a barrier to AHA diffusion and do not influence this process. The amount of acetohydroxamic acid (AHA), *N*(*t*), diffusing over time *t* from a solution to water was calculated by integrating the concentration profile according to the formula:

$$N(t) = S\int\limits_{0}^{\delta } {C_{1} (x,t){\text{d}}x,}$$

where *C*_1_(*x*, *t*) denotes the concentration of AHA at a point situated at a distance *x* from the biofilm–growth medium (LB) interface, *S* is the area of the biofilm–growth medium interface (*S* = 35 × 10^−6^ m^2^), and *δ* is the thickness of the concentration boundary layer (CBL).

The computer program used to analyze these images makes it possible to ascertain concentration profiles and CBL thicknesses. The interferograms produced by the interference of two laser beams are determined by the refraction coefficient of the solute, which in turn depends on the concentration of the substance. If the solute is uniform, interference fringes are straight, and they bend when a concentration gradient appears. The concentration profile *C*(*x*, *t*) is determined by the deviation of the fringes from a straight course. Since concentration (*C*) and the refraction coefficient are assumed to be linear, we obtain:

$$C(x,t) = C_{0} + a\frac{{\lambda {\text{d}}(x,t)}}{hf},$$

where *C*_0_ is the initial substance concentration, *a* the proportionality constant between the concentration and the refraction index (*a* = AHA mol/m^3^ for AHA aqueous solution), *λ* the wavelength of the laser light, *h* the distance between the fringes in the area where they are straight lines, and *f* the thickness of the solution layer in the measurement cuvette. CBL thickness (*δ*) was defined as the distance from the biofilm–LB medium interface to the point at which the deviation (*d*) of the interference fringe from its straight line course is 10 % of the fringe thickness. The membrane system under study consists of two glass cuvettes separated by a horizontally located nucleopore membrane (the place of biofilm formation). After the formation of *P.* *mirabilis* O18 biofilm on nucleopore membrane at 37 °C for 96 h and threefold washing with 0.9 % NaCl, the upper cuvette held water, while the lower one was filled with AHA. A sterile membrane without biofilm was used as a control. The experiment was performed at room temperature. The interferograms were recorded from 120 to 2,400 s (the stationary phase of diffusion was detected by laser interferometry) with a time interval of Δ*t* = 120 s. Such profiles were used to calculate the amount of AHA transported through the membrane as a function of time [*N*(*t*)]. The amount of antibiotic inside the biofilm after time *t* was calculated according to:

$$N(t) = S\int\limits_{0}^{l} {C_{m} (x,t){\text{d}}x,}$$

where *S* and *l* denote the area (7.0 × 10^−5^ m^2^) and thickness of a membrane with biofilm, respectively, and *C*_*m*_(*x*, *t*) is the concentration profile inside the membrane with the biofilm formed. We assumed a linear concentration distribution:

$$C_{m} (x,t) = \frac{{C_{2} (l,t) - C_{1} (0,t)}}{l}\,x,$$

where *C*_1_(0, *t*), *C*_2_(*l*, *t*) are the concentration levels on the two surfaces of the biofilm membrane. *C*_1_(0, *t*), *C*_2_(*l*, *t*) and *l* were determined interferometrically. Membranes after interferometric measurement were stained with FilmTracer (Invitrogen) for membrane coverage confirmation (see section “Analysis of *Proteus mirabilis* O18 biofilm membrane coverage and membrane imaging”).

### Analysis of *Proteus mirabilis* O18 biofilm membrane coverage and membrane imaging

*Proteus mirabilis* O18 biofilm was formed at 37 °C in LB medium for 24–96 h under stationary conditions on sterile nucleopore membranes with a pore diameter of 0.9 μm, which were part of the membrane system of the laser interferometry equipment used for quantitative analysis of acetohydroxamic acid (AHA) diffusion through the biofilm structure. After bacterial growth, nucleopore membranes were washed three times with 2 mL of 0.9 % NaCl and stained with crystal violet (0.4 %) for 15 min. After staining, the samples were washed under the same conditions as above, and the percentage coverage of nucleopore by bacterial biofilm was determined using ImageJ software (Schneider et al. 2012). The membranes were converted to gray-scale digital images with a CanoScan Lide 60 scanner (300 dpi resolution) and saved in TIFF file format. The images were then imported to ImageJ computer imaging software (NIMH, USA). The mean gray levels (1 denotes black color and 256 white) from three membrane images were used to determine the relative gray-scale density of one treatment. CV staining for coverage determination was performed as independent experiment.

After interferometric measurement, biofilm coverage was tested with live/dead staining. The biofilm-covered membrane was washed in sterile 0.9 % saline and stained with FilmTracer (Invitrogen) kit according to the manufacturer’s protocol (O’May et al. 2009). Excess dyes were discarded by washing three times in sterile saline, and the membrane was imaged with a Zeiss Axio Scope.A1 epifluorescent microscope.

### FT-IR spectroscopy

*Proteus mirabilis* O18 was grown in LB medium for 96 h for biofilm formation. Planktonic cells were separated from biofilm and collected. Both samples were freeze-dried. The FT-IR spectra were recorded on a Nicolet 380 FT-IR type spectrophotometer. The sample of bacterial cells and biofilm were placed in direct contact with an infrared attenuated total reflection (ATR) germanium crystal. This technique was used to study the chemical composition of a smooth surface (i.e., biofilms). The FT-IR spectra were recorded from 4,000 to 500 cm^−1^ at resolution of 2 cm^−1^ at room temperature.

## Results and discussion

### Alterations in *Proteus mirabilis* O18 biofilm morphology

**Fig. 1:**
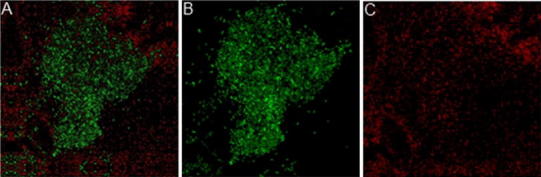
*Proteus mirabilis* O18 pDsRed2 biofilm stained with live/dead BacLight stain (Invitrogen), 72-h culture. **a** Stacked images, **b** live cells, **c** dead cells

**Fig. 2:**
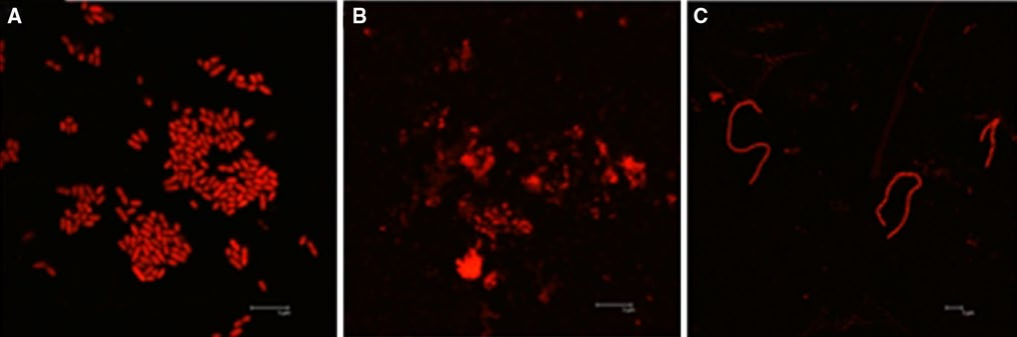
Morphological changes in *Proteus mirabilis* O18 pDsRed2 biofilm after treatment with AHA (**a**), urea (**b**), and both (**c**). Images acquired with a Leica confocal microscope. *Scale bar* 5 μm

*Proteus mirabilis* biofilms are represented by various forms of microcolonies detected by live/dead staining (Fig. 1). *P. mirabilis* O18 carries the pDsRed2 plasmid, which encodes a red fluorescent protein. Preliminary experiments revealed that the light intensity obtained with this technique is lower than that gained with the use of fluorescent dyes. For the best image quality, live/dead staining was used. Assays were performed in at least six repeats, and a representative image was captured and presented. Similar pictures were observed by confocal laser microscopy for living cells only (Stankowska et al. 2012). *P. mirabilis* O18 cells in biofilm were separated by biofilm matrix. Live cells were aggregated in microcolonies; the cells were spread evenly in the field. Dead cells in the microcolonies were found under the live cells (Fig. 1). The untreated biofilm shows a typical structure, where living bacterial cells are separated by exopolysaccharide matrix. The visible layers of live and dead cells were overlaying (Fig. 1a–c). Total fluorescence measurements for *P. mirabilis* O18 pDsRed2 images a, b, and c on Fig. 2 were 22.148, 18.340, and 9.518, respectively. This indicates that in long-cell images, the amount of fluorescent material is reduced to about 57 % as compared to Fig. 2a or b.

**Fig. 3:**
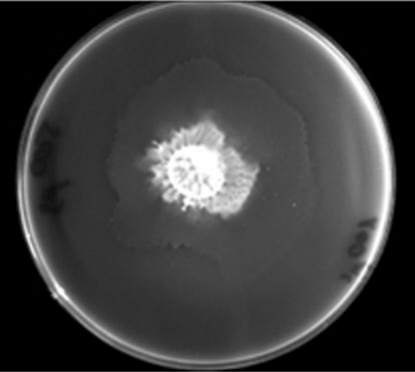
Swarming motility of *Proteus mirabilis* O18 pDsRed in a test plate with LB agar (1 %, w/v) supplemented with AHA (200 μg/mL)

The influence of urea and a urease inhibitor (acetohydroxamic acid, AHA) on *P. mirabilis* O18 biofilm morphology was tested. Supplementation of the medium with 200 μg/mL AHA did not interfere with the morphology of the biofilm. However, supplementation with a high concentration of urea (2 % w/v) did alter the morphology. Changes were visible in the density of cell aggregates and spacing between bacterial cells (Fig. 2b). The use of both urea and a urease inhibitor (AHA) resulted in the presence of a large number of long, undivided cells (Fig. 2c). The majority of the observed cells were in such a long, undivided form. However, some single microcolonies, as in Fig. 2a and b, were also observed. This may be due to the activation of ureolytic activity with a high concentration of urea with simultaneous inhibition of this activity by AHA. One may postulate that this triggers multiple cell functions which result in a swarming phenotype. The presence of swarmer cells in *P. mirabilis* biofilm was also observed by Jones et al. (Jones et al. 2007). However, the role of swarmer cells protruding from biofilm is unclear; some authors suggest that they spread bacteria from the catheter to the urinary tract (Armbruster and Mobley 2012). Bacterial cells in the plates tested for swarming motility showed that the presence of AHA in the medium does not alter this behavior. Swarming motility remains unaltered in the range of AHA concentration from 10 to 1 mg/mL and is similar to that in plates without AHA (Fig. 3). Lack of AHA on *P. mirabilis* O18 pDsRed2 biofilm mass might be due to biofilm extracellular matrix preventing AHA penetration into deeper part of biofilm. That was confirmed by experiment with interferometric analysis (Fig. 8).

**Fig. 4:**
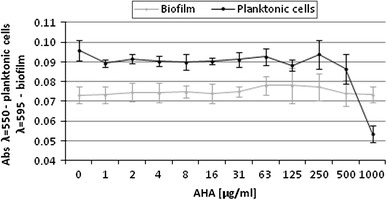
Determination of planktonic cells (absorbance at 550 nm) and biofilm mass (crystal violet method, absorbance at 595 nm) for *Proteus mirabilis* O18 pDsRed strain in increasing concentration of AHA

To determine the kinetics of *P. mirabilis* O18 pDsRed2, culture growth in liquid Christensen medium was conducted for 24 h. It was revealed that the logarithmic phase lasted from 7 to 12 h. In the next experiment, biofilm formation was always started with cells after 8 h of incubation. It was found that the amount of biofilm matrix remains unchanged even at the highest concentrations used (500–1,000 μg/mL). However, the amount of planktonic cells decreased (Fig. 4) in the presence of 1,000 μg/mL AHA. In medical settings, concentrations above 1,000 μg/mL are not used due to AHA toxicity. The results presented in Fig. 4 suggest that concentrations of AHA ranging from 1 to 1,000 μg/mL do not reduce the amount of *P. mirabilis* O18 pDsRed2 biofilm.

Due to its chelating function, AHA can reduce the amount of ions available for bacterial cells. According to Musk and Hergenrother (2008), the presence of AHA affects the process of *P. aeruginosa* biofilm formation by limiting the number of free iron ions present in the medium. It was shown previously that the use of urease inhibitors (acetohydroxamic acid and flurofamide) in urinary tract infections causes a decrease in urine pH and reduces the growth of struvite stones (Stickler 1996).

### Effects of BHL on biofilm urease activity

**Fig. 5:**
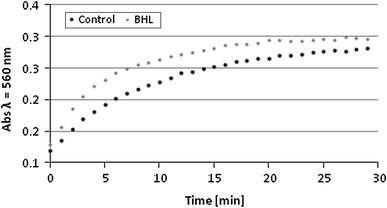
Kinetics of ureolytic activity of *Proteus mirabilis* O18 biofilms treated with 10 nM BHL and control

Previous studies showed that *P.* *mirabilis* lacks an autoinducer type I synthase homolog (LuxI) and probably does not produce this type of autoinducer (Armbruster and Mobley 2012). *N*-acyl homoserine lactone (AHL) does not appear to play a major role in swarming colony biofilm formation (Belas et al. 1998). However, cells react to treatment with *N*-butyryl-dl-homoserine lactone (BHL), and the amount of biofilm increases (Stankowska et al. 2012). BHL treatment has also been found to modify the expression of some virulence factors of *P. mirabilis* strains (Stankowska et al. 2008). In this study, we set out to determine whether BHL affects the ureolytic activity of *P. mirabilis* O18. The influence of BHL on ureolytic activity in *P. mirabilis* O18 biofilm is shown in Fig. 5. The kinetic curves show that the biofilm that was formed in the presence of 10 nM BHL exhibited an increased ureolytic activity by an average of 10.8 % throughout the experiment (30 min). The difference in ureolytic activity was similar in the course of incubation. The obtained data show a statistically significant difference. The mean results and their standard deviations were calculated using one-way ANOVA (*p* < 0.018, *F* = 5.872). Our previous research showed that a 10 nM concentration of BHL increases the biofilm biomass (Stankowska et al. 2012). However, even though the amount of extracellular matrix rises, the availability of urea for cells and their ureolytic activity may not be reduced. Our results suggest that the presence of BHL slightly increases ureolytic activity. Also other studies have proven that different homoserine lactones of various origins can alter biofilm structure and morphology in *Pseudomonas aeruginosa* (Cartagena et al. 2007). The presence of BHL is required for self-production of rhamnolipid surfactants by *P. aeruginosa*, which enable the development of mushroom-like structures in the biofilm (Lequette and Greenberg 2005). Jones et al. (2009) studied the anti-crystalline effect of p-nitrophenyl glycerol (PNPG) and tannic acid (TA). Their results suggest that these substances can inhibit the signaling mechanism of *P. mirabilis* quorum sensing, which implies that TA and PNPG are capable of controlling the growth of crystalline *P. mirabilis* biofilm.

### Urease inhibitor and urea diffusion through *Proteus mirabilis* O18 biofilm

**Fig. 6:**
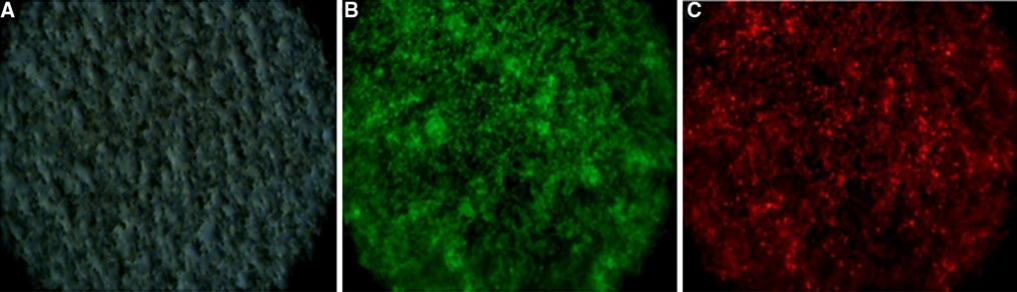
A nucleopore membrane covered with *Proteus mirabilis* O18 biofilm. **a** Membrane surface—light passing through a membrane with round pores, **b** live *Proteus mirabilis* cells on the membrane surface, **c** biofilm stained for dead cells. Live/dead staining was performed with FilmTracer™ dyes (Invitrogen). The images were obtained using a Carl Zeiss Axio Scope.A1 epifluorescent microscope

**Fig. 7:**
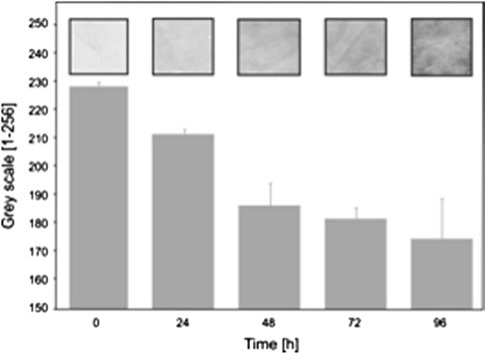
Biofilm area coverage of CV-stained membranes. *Gray* levels from 1 (*black*) to 256 (*white*) represent the levels of coverage, where *high numbers* correspond to low coverage, and *low numbers* to high coverage

Our previous research proved that interferometric measurement of substance diffusion through membranes is a reliable method (Arabski et al. 2009, 2012). In a biofilm diffusion assay, it is essential to cover the membrane with biofilm uniformly. A method suitable for coverage check is staining with a live/dead kit (Film Tracer™, Invitrogen). Our results confirmed that the biofilms covered the membranes uniformly. Figure 6 shows three images: (a) a membrane visualized using the bright field technique, with the round objects being pores in the membrane, (b) live biofilm cells, and (c) dead cells; both images were obtained by the epifluorescence technique. Staining with crystal violet (CV) also confirmed the high coverage of membranes with bacterial biofilm (Fig. 7). It also proved the accumulation of CV-stained materials in a time-depended manner, with uniform coverage after 48 h.

**Fig. 8:**
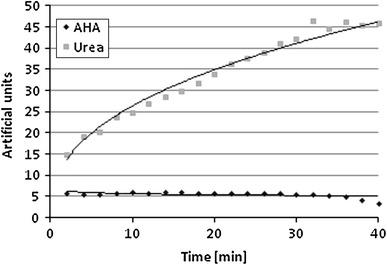
Interferometric analysis of the acetohydroxamic acid (AHA) and urea released from the lower cuvette through a *Proteus mirabilis* O18 biofilm attached to nucleopore membranes at room temperature measured with a laser interferometric system. Representative results are presented. The initial concentration of both substances was 250 μg/mL

Interferometric analysis of AHA diffusion through *P. mirabilis* O18 biofilms attached to membranes shows that only up to 4.5 % of AHA concentration emerges on the other side of the membranes (data not shown). This suggests that AHA concentration in biofilm may decrease by more than 20 times. *P. mirabilis* O18 biofilm constitutes a firm barrier to this urease inhibitor. In contrast to AHA, urea diffuses readily (Fig. 8). In terms of cell layers, the deeper regions of biofilm are protected from AHA inhibition. On the other hand, urea continues to be supplied and is a source of carbon and nitrogen. The dramatic reduction in AHA diffusion might be due to AHA binding to heteropolymeric biofilm structures, while the protonation of urea allows it to diffuse through the membrane in an undisturbed manner. Other studies have also revealed problems with drug delivery in urease-associated infections (Umamaheswari et al. 2002).

**Fig. 9:**
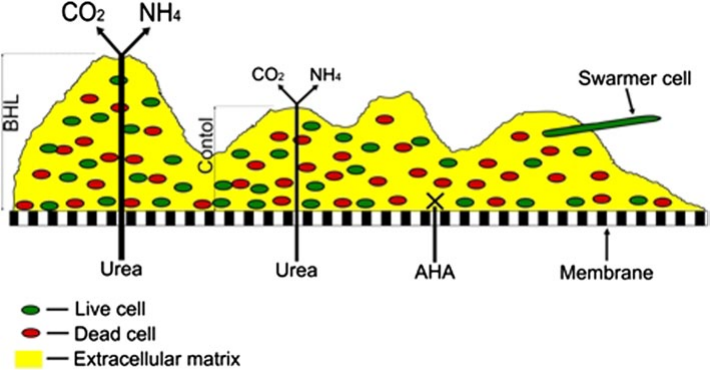
Scheme visualizing the obtained data. The addition of 10 nM BHL increased both biofilm thickness and its ureolytic activity. Diffusion of the urease inhibitor is blocked, while urea diffuses freely. Starvation conditions may induce the presence of protruding swarmer cells. Biofilm mass tightly covers the nucleopore membrane

In conclusion, the presence of *P. mirabilis* O18 biofilm is a very efficient barrier to AHA diffusion. Under the same conditions, the diffusion of urea is not prevented. This suggests that the deeper regions of biofilm are protected against the urease inhibitor, while *P. mirabilis* cells still have access to urea, which is a source of nitrogen and carbon. The inhibition of ureolytic activity also revealed the presence of long cells. The swarming behavior of *P. mirabilis* O18 biofilm cells is probably triggered by starvation conditions. Previous studies have shown that the presence of BHL increases biofilm thickness and its ureolytic activity. A model developed on the basis of the obtained results is presented in Fig. 9.

### Spectroscopic studies

**Fig. 10:**
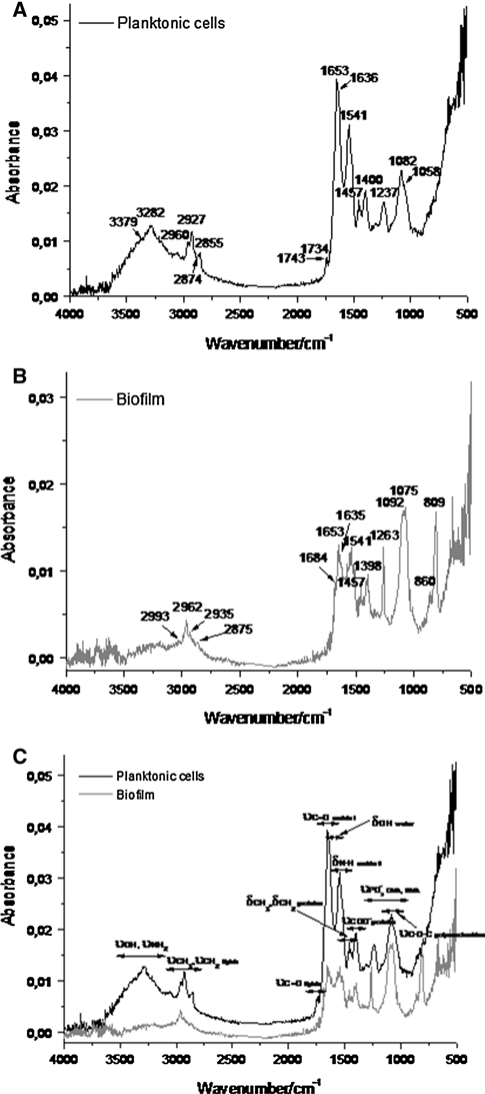
Representative FT-IR spectra (4,000–800 cm^−1^) of *Proteus mirabilis* planktonic cells (**a**), *Proteus mirabilis* biofilm, (**b**) and a superposition of the above-mentioned cultures (**c**)

Experimental FT-IR spectra, mainly in the region of 4,000–500 cm^−1^, are very useful for the identification of different functional groups of amino acids, fatty acids, polysaccharides, and inorganic anions and provide further information on their biological structure. Figure 10c juxtaposes representative FT-IR spectra of *P. mirabilis* O18 planktonic cells and biofilm. The main features of these spectra are quite informative, and they offer an insight into differences between the planktonic and biofilm forms of *P. mirabilis* bacteria. The first typical bacterial spectrum (Fig. 10a) is mainly a superposition of the IR fingerprints of five types of biomolecules: water, proteins, lipids, polysaccharides, and nucleic acids. The very broad band at 3,379 cm^−1^ is due to hydrogen-bonded O–H groups. Moreover, the presence of water molecules confirms the bending mode at 1,636 cm^−1^. This is also linked to stretching vibrations of the NH_2_ moiety in proteins (3,282 cm^−1^). Amino acids were identified by the vibrations of the peptide linkage, and especially the amide I band (1,653 cm^−1^) arising from backbone amide carbonyl νC=O vibrations and the amide II band (1,541 cm^−1^) arising from the out-of-phase combination of N–H in-plane bending vibrations and C–N stretching vibrations. The CH_3_ and CH_2_ deformations associated with proteins (1,457 and 1,400 cm^−1^) are also apparent. In addition to the protein absorption bands, asymmetric and symmetric νCOO^−^ vibrations are present in this region (1,653 and 1,400 cm^−^1). The absorption peaks at 2,960, 2,927, and 2,855 cm^−1^ are mainly due to the asymmetric and symmetric stretching of methyl and methylene groups in bacterial cell wall fatty acids and other components. The FT-IR spectral pattern of planktonic cells shows C=O stretching vibrations (1,734 cm^−1^), which were attributed to ester functional groups in lipids and fatty acids. In turn, polysaccharides are localized mainly in the region of 1,200–900 cm^−1^. The very strong band at 1,082 cm^−1^ is assigned to C–O–C stretching vibrations and corresponds to the sugar skeleton. The same region consists of the vibrational features of DNA and RNA. These bands are associated mainly with the symmetric and asymmetric νPO_2_^−^ stretches (1,237 and 1,058 cm^−1^) of phosphate groups. The most significant differences between the spectral features of planktonic cells and biofilm are mainly due to νCH_3_, νCH_2_, νPO_2_^−^, and νC–O–C stretches (Fig 10b). In the FT-IR spectra of *P. mirabilis* biofilm, strong bands attributed to the presence of methyl and methylene stretching vibrations are observed at 2,993, 2,962, and 2,875 cm^−1^. Moreover, the broad band in the 3,300–3,100 cm^−1^ region, found in planktonic cells, disappears in the infrared spectra of the biofilm. Other variations due to the C–O–C stretching vibrations of polysaccharides (1,092 cm^−1^) are apparent. Differences between planktonic cells and biofilm can also be seen in the P=O bonds of the phosphodiester backbone of DNA and RNA, which are up-shifted by 16 and 17 cm^−1^, respectively. This is probably caused by the different spatial DNA structure of planktonic cells and biofilm. Interestingly, a new strong and intense peak appears at 809 cm^−1^ for *P. mirabilis* biofilm. This spectral feature may result from the involvement of nucleic acids and polysaccharides in extracellular matrix formation.

## Conclusion

*Proteus mirabilis* O18 biofilm is a nearly impenetrable barrier to AHA, but it does not inhibit the diffusion of urea. This suggests that deeper biofilm regions are protected from the urease inhibitor, while bacterial cells still have access to urea, a source of nitrogen and carbon. Spectroscopic studies suggest that biofilm matrix is composed not only of polysaccharide polymers but also of nucleic acids, which may be involved in biofilm structure formation. The inhibition of ureolytic activity also revealed the presence of long cells, whose role is still unclear. The swarming behavior of *P. mirabilis* O18 biofilm cells was probably triggered by starvation. As previous studies have shown, the presence of BHL increases biofilm thickness (Stankowska et al. 2012) and its ureolytic activity. The presented data may suggest that the use of a urease inhibitor in CAUTIs may be less effective than in other urease-associated infections.
